# The experience of adolescents participating in a randomised clinical trial in the field of mental health: a qualitative study

**DOI:** 10.1186/s13063-016-1474-2

**Published:** 2016-07-28

**Authors:** Nick Midgley, Danny Isaacs, Katharina Weitkamp, Mary Target

**Affiliations:** 1 Anna Freud National Centre for Children and Families, London, UK; 2 Research Department of Clinical, Educational and Health Psychology, University College London, London, UK; 3 MSH Medical School Hamburg, Hamburg, Germany

**Keywords:** Randomised controlled trials, Trial participation, Patient understanding, Patient perspective, Parents, Adolescence, Depression, Qualitative research, Framework analysis

## Abstract

**Background:**

This descriptive study aimed to investigate adolescents’ motivations for participating in a randomised controlled trial (RCT), to explore the understanding that the young people had regarding a number of aspects of the trial design, to examine whether or not they found participation in the trial to be acceptable and what affected this, and to identify whether and how the young people felt that their participation in the RCT impacted on their experience of therapy and on therapeutic change.

**Methods:**

Seventy-six adolescents who were taking part in a large-scale RCT to evaluate the clinical and cost effectiveness of psychological therapies for depression were interviewed at two time-points after completing therapy. The semi-structured interviews, which included a focus on the young people’s experience of the research study, were analysed using framework analysis.

**Results:**

The vast majority of adolescents found it acceptable to participate in the clinical trial, and many agreed to participate for reasons of ‘conditional altruism’. However consent was often given without great understanding of the key elements of the trial, including the difference between treatment arms and the randomisation process. Although the adolescents were largely positive about their experiences from taking part, the study raises questions about whether clinical outcomes may be influenced by participation in the research elements of the trial.

**Conclusions:**

Although adolescents are under-represented in clinical trials, those who do participate are generally positive about the experience; however, careful thought needs to be given to key elements of the trial design and the potential impact of the research participation on clinical outcomes.

**Trial registration:**

ISRCTN registry, ISRCTN83033550. Registered on 15 October 2009.

## Background

In the most recent edition of their comprehensive review of ‘What works for whom?’ in the field of child and adolescent mental health, Fonagy et al. [1] expressed the view that randomised controlled trials (RCTs) are ‘generally considered scientifically superior to cohort or observational studies in answering the question of whether a treatment is effective’ (p. 4). Although these authors acknowledge the limitations of RCTs, this opinion is shared by most developers of guidelines on evidence-based practice such as those developed by the American Psychiatric Association in the USA or the National Institute for Health and Clinical Evidence in the UK.

Yet despite the importance of participation by adolescents, relatively little is known regarding how and why young people decide to participate in clinical trials, especially in the field of mental health, or how they then experience involvement in such studies [2]. A recent study by Brown et al. [3] noted that adolescents are under-represented in clinical trials generally and that ‘little is known about adolescents’ knowledge and attitudes surrounding clinical trials’ (p. 213). Brown et al.’s own questionnaire survey of 82 high school students in Michigan found that only 33 % of those surveyed had ever heard of a clinical trial, and understanding about trials was poor. However, attitudes toward taking part were somewhat more positive.

The literature examining the experience of *adults* who have participated in clinical trials is somewhat more extensive. In a systematic review article by O’Cathain et al. [2], 54 studies were identified which had examined the design, process and conduct of clinical trials using qualitative methodologies. Among these were studies that examined how and why participants decided to take part in clinical trials, their understanding of trial design and whether trial design was considered acceptable to those who took part.

Most research examining the views of adults taking part in clinical trials has focused on studies evaluating medical treatments. However, Notley et al. [4], in a well-designed study examining the views of 13 young adults on their involvement in a trial of social recovery cognitive-behavioural therapy, found that practicalities relating to participation, such as the time and location of research meetings and flexibility in relation to these from the research staff, were important factors in the patient’s experience of taking part. Participants also expressed positive surprise at their levels of disclosure in research assessment meetings, and stated that this might have happened as a result of the researcher being supportive, non-judgmental and empathetic. Taken together, these findings may suggest that the patients’ experience of participation in a research trial is acceptable if they experience research staff in such positive ways and if they feel that they are getting something positive from the experience of taking part in the trial.

When it comes to adolescents, a certain amount of literature exists in the field of *physical* health—especially oncology—regarding the recruitment and decision-making process as to whether to participate in research or not, from the perspective of both adolescents and their parents (e.g. [5–8]). Very few studies, however, have focused on the experience of adolescents *after* they have chosen to participate in a trial, and research related to young people’s involvement in clinical trials evaluating the effectiveness of psychological therapies is completely lacking.

In conclusion, whilst a reasonable amount of knowledge is available about the experience of *adults* who have taken part in clinical trials in the field of medicine, and a few studies that have looked at the experience of those taking part in trials evaluating psychological therapies, no research is available that explored the experience of adolescents participating in clinical trials of psychological therapies, and relatively little research is available on young people's participation in clinical trials generally. Where such research has taken place, it has mostly focused on the decision to participate, with less attention paid to the experience of actually taking part in a trial. But exploring such experiences is important for two key reasons. First, it is essential to know whether the core elements of clinical trials (recruitment, randomisation, and on-going data collection) are understood and found acceptable or not to adolescents. Such information is crucial to inform the design of future clinical trials in a way that is ethical for those being asked to participate. Second, given the debates about the transferability of trial findings to the routine clinical practice [9], it is important to understand, from the perspective of the young people themselves, whether participating in the trial impacted on the treatment they received. Was the therapeutic impact of the intervention different because it was taking place in the context of a clinical trial?

The aims of this study, therefore, were (1) to explore adolescents’ understanding and motivations for participating in an RCT investigating the therapeutic efficacy of three treatments for adolescent depression, (2) to explore whether or not the young people found participation in the trial to be acceptable and what affected this, and (3) to explore whether and how the young people felt that their participation in the RCT impacted on their experience of therapy and upon therapeutic change.

## Methods

### Setting for the study

All the adolescents interviewed for this study were taking part in a large, multi-centre, randomised controlled trial, the Improving Mood through Psychoanalytic and Cognitive-Behavioural Therapy (IMPACT) Study (for full details of the study, see [10]). IMPACT is a pragmatic superiority trial comparing the relative clinical effectiveness of three psychological treatments for adolescent depression, with established evidence of efficacy for evoking clinical remission in the short term (i.e. 3–6 months). The three treatment approaches tested in this study were a manualised form of specialist clinical care termed brief psychosocial intervention (BPI), short-term psychoanalytic psychotherapy (STPP) and cognitive behavioural therapy (CBT).

### Participants and recruitment

Potential participants in the IMPACT trial were identified by clinical staff from routine referrals to the participating Child and Adolescent Mental Health Service (CAMHS) clinics. The assessing clinicians informed the young person and the parents/carers about the trial and invited them to consider taking part. They were told that the study team would be in touch if they expressed an interest to participate, and that recruitment would depend on research assessments of whether the patient met the inclusion and the exclusion criteria.

The participants and their parents or legal guardians were then sent information sheets about the trial and were asked if they were willing to be contacted by a researcher, who then met participants and invited them to sign a consent form. In agreeing to participate in this pragmatic clinical trial, the young people were agreeing to be randomised to one of the three treatment arms. As the trial was examining levels of relapse in the medium term, participants were also agreeing to attend six research assessment meetings across an 86-week period, in each of which they and their parents or carers were asked to complete a substantial number of structured assessments. These assessments were a mixture of structured interviews and self-report questionnaires on various aspects of their psychological well-being, and usually took approximately 1.5 hours to complete (for full details, see [10]). The information sheet explained that young people would be offered a £10 voucher for attending each assessment meeting (see [10]). Following randomisation, all patients in the trial were treated by appropriately trained and supervised CAMHS staff working in the participating clinics.

A total of 465 young people were recruited to the IMPACT study, with a mean age of 15.6 years (SD 1.4). A total of 348 (75 %) were females, and 117 male (25 %). Of these participants, 85 % (382/450) described their ethnicity as White British. At the point at which the young people had been recruited to the study, all met the diagnostic criteria for moderate to severe depression, based on the Kiddie-SADS [11]. The IMPACT study took place across different parts of the UK; the recruitment numbers were 127 for North London, 185 for East Anglia and 153 for the North-West (Manchester and the Wirral). Findings regarding the comparative clinical and cost effectiveness of the interventions are expected in 2016.

Alongside the IMPACT study, a sub-study known as IMPACT-ME [12] aimed to explore the experience of the participants taking part in the London arm of the IMPACT trial (for other findings from this study, see [13–15]). Recruitment for this sub-study began slightly later than the main clinical trial, in September 2011, with all young people recruited in London being asked for additional consent to take part in two semi-structured interviews taking place 36 weeks and 86 weeks after they had joined the IMPACT trial. The participants for the sub-study reported in this paper, therefore, were 76 adolescents who were recruited to the London arm of the IMPACT study between September 2011 and December 2012 and who completed at least one of the two post-therapy semi-structured interviews. Closely matching the total sample in the IMPACT trial, the average age of these 76 young people was 15.35 years, and 76 % were female. Based on a review of all key characteristics, this sub-sample was broadly representative of the participants in the London arm of the trial itself.

### Data collection

For the study reported here, a qualitative approach was taken, as the aim was to allow the young people to describe in depth their experience of participating in the trial, as well as their views of the psychological therapy and their understanding of any changes in their depression that took place during and after therapy. We chose qualitative research, because it aims to describe and understand social or human phenomena. The research process is reflexive and interactive and allows the participants to express their experiences in their own words. Verbal or visual data is interpreted based on distinct methodological traditions to get a complex holistic picture [16]. In the current study, young people were interviewed using a semi-structured protocol, the *Experience of Therapy Interview* (ETI) (Midgley N, Ansaldo F, Parkinson S, Holmes J, Stapley E, Target M: Expectations of therapy interview (young person and parent versions), unpublished). The interview schedule covered three key areas: the difficulties that brought the young person into therapy, the participant’s experience of therapy, and how it had been to participate in the IMPACT study.

Interviews were conducted on two occasions: at the end of therapy (36 weeks) and again at 1-year follow-up (86 weeks). These interviews were carried out at a different time from the research assessment meetings for the IMPACT trial itself and by a different team of researchers (this was done primarily so that the research assistants working on the IMPACT trial would not be un-blinded to the treatment allocation, given that the qualitative interviews included an in-depth exploration of the young person’s experience of therapy; it also aimed to make it easier for the young people to speak openly about their experience of involvement in the trial, including both positive and negative aspects). In line with guidance on qualitative interviewing, researchers were encouraged to follow the young person’s lead, but follow up with further questions in order to gain as deep an understanding as possible of their experience. All interviews for this sub-study were carried out by post-graduate psychologists, who were given a half-day training session in semi-structured interviewing using the ETI, and were offered feedback on interviewing technique following their initial interviews with young people.

Interviews were audio-recorded with interviewee consent. The interviews at 36 weeks took place between October 2011 and January 2014, and the interviews at 86 weeks between August 2012 and January 2015; 70.5 % of the young people had interviews at both time points. All interviews took place in the young person's home or at their local CAMHS, depending on the young person’s preference.

### Data analysis

All interviews were transcribed and then analysed using framework analysis (FA) [17], a method of qualitative data analysis that was originally developed in the context of social policy research in the UK but has recently become increasingly popular in the fields of nursing and psychology. Sitting within the family of broadly ‘thematic’ approaches, FA provides a flexible but structured approach to data management and data analysis, which is especially suitable for studies which have quite focused research questions, a relatively large amount of qualitative data that needs to be managed, and a priori issues to investigate [18]. It also lends itself to studies where qualitative data analysis is carried out by a team working together, rather than an individual researcher. The approach has also been integrated with the NVivo10 qualitative software package [19], which was used in this study.

We followed the five stages of framework analysis, as outlined by Ritchie and Spencer [17]. Two of the authors (DI and KW) developed a coding framework focusing on the main research questions, under the supervision of NM and MT. They coded the first 20 interviews together with the aim to develop a comprehensive, consistent and clear coding framework. The first author then coded a number of additional interviews using this framework, to ensure that it was sufficient, comprehensive and reliable. Further revisions were made to the coding framework based on this process, and then, DI and KW continued to code the remaining interviews using the coding framework. In addition to the inductive coding based on the framework, each case was coded at a global level in terms of main motivation for participation, general acceptability of participation, and the overall understanding/lack of understanding of participating in the RCT. Based on these ‘global’ ratings, frequencies will be reported in the results section. (A more detailed account of the use of framework analysis in this study can be found in [20]).

## Results

Findings are presented in three sections, following the three research aims of the study. Each section is reported in a narrative form, but numbers are given to indicate how common each theme was. However, not all participants made reference to experiences that related to every category within the framework, and in some cases, data from one interview could be coded to several categories (for example, when a young person spoke about several reasons for choosing to participate in the study); therefore, the total number of young people reported for each category does not always add up to 76. In our findings, the terms ‘young people’ and ‘adolescents’ are used interchangeably.

### Young people’s understanding and motivations for participating in the RCT

Although the semi-structured interview did not set out to systematically assess the level of understanding of the trial design, the way young people spoke about the IMPACT study revealed their various understandings of different elements of the research, including the study objectives, key features of the design such as randomisation, and the relationship between treatment and research. Overall, 34/76 participants demonstrated an understanding of the study, compared to 33/76 who seemed to have a general lack of understanding. One young person expressed a lack of understanding in the following way:

*I probably didn’t understand it as well as I should have. I don’t even know the one I’m doing now, it’s really bad. Because I was reading them and they all kind of sounded the same to me, apart from one with antidepressants or something. I didn’t really know the differences, I was just thinking I’m getting therapy, that’s that. (female, 16)*

Whilst some participants demonstrated their understanding that the study was aiming to help young people with depression, others showed a more detailed understanding that the study aimed to compare therapeutic efficacy of three different types of treatment:

*As far as I know it’s to do with working out what type of therapy works best for teenagers or people, I think it’s from 11 to 16 or something. Cos there was Cognitive Be- there was cognitive therapy, psychotherapy…um, one that was a bit more like counselling…and I think there was one other one. And then like we all got picked one at random and then…they checked – so they monitored us to see if we were getting better and then, from that they’d do something sciency and then work out which one’s best. (female, 14)*

Despite the fact that approximately half of the young people did not seem to fully understand what the trial was about, these participants were nevertheless able to offer a number of different reasons why they had agreed to participate in the study. Of all those mentioned, helping others or contributing to understanding of depression was the most common, with 36/76 participants raising this:

*Well, I don’t know how much I’m helping but just knowing, just knowing that even if I contribute by one percent in helping another individual then I think that’s just, it’s better than zero percent. (female, 16)*

Thirteen of 76 young people spoke about their participation in the study in relation to getting treatment or help. Although it was made clear that they would receive an intervention whether or not they chose to participate in the trial, these adolescents described how they believed that taking part in the study would lead them to getting help or treatment. More specifically, three young people spoke of motivation to take part in the study due to a belief that they would getter *better* help or treatment, as everything would have to be *‘done by the books’*. For one participant, the prestige of the study seemed to influence how the participant perceived the treatment to be received:

*It just sounded like it sounded like bigger, like more successful, and…that it might actually be able to help me. (female, 16)*

Furthermore, five young people stated that they participated in the study because they believed it would offer them quicker access to treatment or help:

*He offered me IMPACT or he was like ‘well you can have normal therapy but you would have - but then you would be put onto a waiting list’…So I was like, yeah I’ll do IMPACT. (female, 17)*

Eleven young people spoke about taking part in the study because the research itself *‘sounded really interesting’ (female, 15).* Whereas one young person vehemently stated that she did not think young people should be paid for taking part, five others spoke of the money they were paid for taking part as an important motivator.

*I like the idea they pay you for it. I’m not gonna lie (laughs). It’s really – it’s a very good incentive. (female, 17)*

Six participants spoke about how they liked the opportunity to be part of the research because it made them feel less isolated. One young person spoke specifically about the other people in the study and gave the impression that knowing that other young people were in the same position as her was in some way reassuring:

*It’s weird to think that so many other people over the country are, like you know — people are coming to their house as well, and they’re getting interviewed and like spoken to. (female, 16)*

### The acceptability to young people of taking part in the trial

Based on their interviews, 71/76 (93 %) of the participants found participation to be generally acceptable, with only four finding participation to be generally unacceptable. One participant was fairly typical of this positive view, when she said:

*I think it’s been like quite a good experience… it feels interesting I suppose to- to know that like you’re kind of part of the study (female, 16)*

More specifically, 21 young people talked about how taking part in the study—in particular the regular research assessment meetings—had helped them to see the progress that they had made:

*Yeah, it was a really good way. And although I thought like, ‘Oh, you know like, why do they have to come. Like I’m better now’. But when they got here, and I actually started like talking to them and like doing the things I thought of…it felt good. It’s like you really can see the progress you’ve made. (female, 16)*

Despite the overall sense that participating in the trial had been acceptable, 40 participants spoke about specific aspects that they had found less acceptable. The questionnaires in particular seem to have been experienced as too time consuming. Young people stated that the questions were often repetitive, felt irrelevant, completing the questionnaires was tedious, and that it was difficult to focus on completing the questionnaires because of the length. Furthermore, some felt that the questionnaires were unreliable, as the questions often asked about how one had been feeling within a specific time-period, and they did not feel it was possible to answer such questions accurately or meaningfully. Some participants also reported finding the interviews and the questions asked somewhat intrusive or invasive, for example questions about suicide. This participant was fairly typical when he responded to a question about how he felt the research should have been done differently:

*Make [the research assessment meetings] not as long. They’re quite long and there’s lots of questionnaires and sometimes it’s difficult to answer everything right cos you’re just kind of ticking off stuff… Because sometimes you just – it gets to the point where you’re just like ‘ok, getting a bit tired of this now’ and then you don’t feel like what you’re saying is particularly useful because it’s not* not *true but not –you’re just saying things as quickly as possible cos you wanna stop. (male, 16)*

On the other hand, 23 participants found completing the questionnaires to be manageable, particularly as they progressed through the study. (The number of measures used in the study was reduced at later assessment points, in the light of negative feedback from participants about the baseline assessments). In addition, a few young people reported that completing the questionnaires had been helpful, enabling one to see a wider range of perspectives or views, helping one to see change and encouraging one to think and examine their difficulties and develop a better understanding.

*There’s like loads of questions but like it really makes you think about stuff and it really like examines everything and even though I don’t like examining… it’s kind of like, I dunno, it felt like more helpful - like I thought about things that could be related, that wasn’t like my phobias and stuff and I was just like… it definitely - it definitely helped me understand myself a bit more. (female, 16)*

Just under half (35/76) of the young people spoke about the process of randomisation, and of these, 15 showed an understanding of the randomisation process, most commonly explaining that randomisation made things fair:

*I think it’s a good method… It’s not biased or anything so, yeah, so that’s better, randomly allocating them. (female, 15)*

In comparison, 20 participants seemed to have a lack of understanding or, in a few cases, explicit misunderstanding about randomisation. Some had a difficult time remembering the randomisation process, and one questioned whether it had actually taken place. Most commonly, the belief was expressed that within randomisation, the professionals would have chosen the treatment that was best for their individual needs. This was stated explicitly by seven young people:

*I just thought well they would know what, which treatment would be best for me so I didn’t really think about it that much. (female, 16)*

For 14 participants, being randomised to a treatment was a source of concern for them regarding their participation in the trial. Anxieties about not knowing which therapy one will get, a feeling of a lack of information about each therapy leading to anxiety about being allocated a *‘bad’* one, or one that would not suit their needs or work, or believing that some were not even designed to treat depression were present in the narratives of these young people. They expressed that it would have been better if choice was given rather than random allocation; choice based on knowledge of the young person and their needs. One young person described feeling *‘annoyed’* at the possibility that there could have been a therapy better suited to them:

*I think maybe the random allocated way about it isn’t really the right way to go cos there’s obviously some people with worse issues than others, so if there’s different things that you can choose from, why would you randomly put them in, would you not want to kinda look at their background or ‘this would be better for that person, this would be better for that person’? (female, 15)*

Furthermore, 11 young people were unhappy about the outcome of the randomisation process, stating that they would have preferred a different treatment arm as they felt it would have been more helpful. Others expressed a wish to try all three to see which would work best for them. However, very few young people seemed to have a clear understanding of what the three different treatment options involved. For one young person, the idea that the three possible treatments were different seemed to mean that they were not all treatments for depression; however, when this was explained to the participant by a research assistant, the participant seemed to gain greater understanding about the relative aims of each treatment:

*Oh no it was, it was before they actually told me about it ‘cause I thought they were, like I knew they were the all the same thing****,****but I didn’t realise they were helping for the same thing. ‘Cause when she said it was three different treatments****,****I thought they were completely different, but when she explained it, I realised they were all actually related, and to do with the same thing. (female, 16)*

Thirty-eight of the participants spoke about finding it difficult to remember the differences between the treatment options, and perhaps most significantly, when they could remember that there were three options, they reported that they all sounded the same or that it was difficult to know what the difference was between them. When asked how they felt about the randomisation process, one young person stated:

*I probably didn’t understand it as well as I should have. I don’t even know the one I’m doing now, it’s really bad. Because I was reading them and they all kind of sounded the same to me, apart from one with antidepressants or something. I didn’t really know the differences, I was just thinking I’m getting therapy, that’s that. (female, 16)*

Despite not fully understanding how the treatment options differed, 22 young people seemed to find the randomisation process fairly acceptable, particularly once they had been reassured that all three treatment arms were expected to be effective. In addition, they expressed that they were happy to be getting help, regardless of the treatment arm to which they were allocated, or that their lack of experience and knowledge about therapy meant that it would be better to *‘leave it to the experts’*. The idea that randomisation was a *‘fair’* and *‘not biased’* way to decide which therapy each young person received was also expressed. In contrast to those unhappy about the outcome, 22 others seemed to be happy about the treatment allocated to them. These young people seemed to feel that they had been given *‘the correct’* treatment or the one they were hoping to get, or that any of the treatments would have been helpful.

Of all the elements of the research, the topic that participants spoke most often about was the meeting with the research assistants. Fifty-five of 76 of the young people reported positive experiences with the research assistants, which seemed to contribute to acceptability of participation. Research assistants were described as friendly, nice, easy to talk to, not judgemental, polite and interested. Participants reflected that flexibility from the research assistants in terms of location and timing of meetings and the questions asked during the meetings made them feel more comfortable and enabled them to talk. They also described that it felt more like they were talking to a peer, that is, more normal and less formal, perhaps because the research assistants were closer in age than their therapists:

*They’d come to me so I was at my house, so I felt more comfortable. Rather than going to someone that I don’t really know, and going to a place that I’ve never been to before, you just felt more comfortable…and if I wanted to change to different question we could, and then we could come back to it. (female, 15)*

However, 14 participants reported less positive experiences of the meetings with the research assistants. It was described as *‘weird’*, *‘strange’*, and *‘scary’* to have the research assistants come to the young person’s house and for the participant to tell this new person about their difficulties. For one young person it also felt *‘heavy’* to be asked questions about their difficulties, and another described disliking the approach that a research assistant took in persisting with a line of questioning, when the participant had given an answer.

The nature of the study was such that different research assistants met with the adolescents over time. Twenty-one of the participants reflected that they did not mind changes in the research assistants to whom they spoke. For some, not seeing previous research assistants again meant that they could talk more openly; for others, the feeling was expressed that changes in the research assistants did not matter, as they were not there to develop close relationships but to ask questions. However, 26 young people seemed to value the consistency in the research assistants they met with and found inconsistency to be more unacceptable. Having to start all over again with a new person seemed to make it more difficult and less comfortable for the young people to talk about their experiences:

*To see so many different faces and to explain your story over and over and over again it is hard, it is difficult… I don’t know how many people I’ve had - I’ve had well over 20 people come to this house and see me and I’ve had to explain my story every single time. (female, 14)*

For 14 participants, the fact that the therapy and the research meetings were tape recorded was described as *‘weird’* or *‘awkward’*. Young people seemed to feel conscious of the recorder and the fact that there would be a record of what they were saying. This seems to have contributed to a feeling that it was *‘scary’* to be tape recorded. However, 29 participants stated that they got used to the tape recorder over time, and that it did not seem to be something that bothered them or that they thought too much about:

*It wasn’t too bad… I mean I didn’t really pay attention to the recorder that much… I mean I would occasionally look at it but I wouldn’t really… be wary of what I’m saying knowing that I’m being recorded. (male, 17)*

### How participating in the trial impacted on the experience of therapy and therapeutic change

During their interviews, 40/76 participants spoke about the relationship between the therapy they were receiving and the study itself. This was predominantly in an indirect manner, whilst describing how they thought of their therapy and their participation in research meetings. Of these, 15 young people seemed to perceive research and therapy to be one and the same or to have a significant amount of confusion or lack of clarity over the differences between research and therapy. Quite often this was demonstrated in the way they used the term ‘IMPACT’ to refer both to the therapy they received and the study itself. In some cases, the interview process itself made them aware of their confusion, as in the following exchange:

***I: How did you feel about those [research assessment] meetings…***

*P: They were alright… I thought they were just part of the therapy…*

***I: Right okay…***

*P: Well not part of the - cos they did ask me about my depression and stuff*

***I: Yeah…***

*P: Wait, are you the same people? (male, 17)*

The other 25 participants who spoke about the relationship between the treatment and research did appear to have an idea that they were distinct from one another, but how they saw this distinction was often quite varied. As one put it:

*Cos obviously it’s not exactly the same as being in a room with your therapist, I mean it’s not exactly the same. I mean with these researchers you can say how your moods and stuff are, but with your therapist you can say like what has actually gone on altogether. (male, 15)*

Interestingly, some of these young people could see similarities between the research and therapy even though, fundamentally, they knew that they were distinct things:

*It almost feels like they’re the same thing but…they’re not […] Well the research is much more direct. I’m asked questions as opposed to I say what I want to say and sometimes it’s a bit more intense than just sitting there talking to someone. But it’s – it feels just fairly similar. (male, 16)*

Of the 32 participants who spoke about the relationship between therapy and research meetings, 24 stated that they preferred the research assessment meetings. Amongst the reasons for this were the flexibility in having research meetings at home; questionnaires making one think about and examine things to a greater extent than therapy; a feeling that research assistants were easier to relate to, e.g. being closer in age, ‘friendlier’ and ‘more understanding’ than the professionals in CAMHS; and the meetings with researchers were ‘less formal’:

*I think I enjoyed [the meetings with the research assistant] more than the weekly therapist’s. Because she’s really nice and she’s more near my age and I think that’s why I feel more comfortable with her, because we kind of talk about the same kind of stuff and almost have the same kind of interests and I think it’s easier when you’re talking to someone who’s close to your age, than someone who’s a lot older because they’ll understand more. So I felt a bit more comfortable with [the researcher]. (female, 16)*

On the other hand, eight participants stated that they preferred therapy over research. Amongst the reasons for this was the feeling that the therapist actually listened as opposed to the researchers who just asked questions, the feeling that therapy was more useful, and that participants could speak about what they wanted to speak about. For some young people, the fact that therapy seemed to be more formal—which others had found a negative point—helped them to trust the therapist more than the research assistants:

*With therapy I think it was more on a formal level but then it was satisfying cos I could talk to her about anything that I really wanted to, but like I trusted her more the person more than like with the research. (female, 15)*

Thirty-one participants spoke about a range of ways in which participating in the study and having regular research assessment meetings impacted on the outcome of therapy and in recovery from depression. Four spoke about how participating in the study gave them the sense of being monitored or followed up on and that this was helpful, regardless of whether they found the therapy helpful or not.

*I’ve really, really liked it, I mean just… having somebody have a little check up with you and knowing that… for instance although therapy didn’t work for me, I’m pretty sure that if I started feeling really bad again like IMPACT - I felt as if I could trust them that they would try and like sort something out for me. It’s just they’ve kinda always been in the background. (female, 17)*

Three young people spoke about how being part of the study alongside therapy gave them the sense that they were not alone in their depression and that others must be going through similar difficulties.

*Like I think once you guys sent me this sheet thing and it had all these like facts on it, and it was like how many people are in the study. And I couldn’t believe it. Cos obviously like me, like I know – well, I don’t know for sure – but I’m sure most of the people that I know, that I’m around, aren’t going through, or haven’t been through anything. So I just think I’m a bit of the odd one out. But then knowing that so many other people in the country that are going through it, it makes it makes you feel a little bit less…weird. (female, 16)*

Three young people also spoke about how participating in the study helped them to attend all therapy sessions either as a result of the knowledge that therapy was time-limited or because it helped them to become aware of the progress that they were making:

*I think because of the study there’s more structure to it, like I knew that it was only gonna be like 20 [sessions], and I knew when I’d be meet - there was just more, it was just more structured so I knew like how long I had… (female, 16)*

There was a range of other ways in which adolescents felt that participating in the study had positively impacted on the outcome of their depression. Fifteen young people reported finding the opportunity to talk about their difficult experiences in the research assessment meetings as a positive or helpful aspect of the study:

*I mean these little sessions were helpful in a way that I could…talk about most things, whereas I don’t really talk about any of this stuff with anyone else … they were helpful in that sort of way. (male, 17)*

On the other hand, 24 young people felt that participation in the study did not have an effect on the outcome of their therapy. Eleven of these stated that they simply felt that participation did not have an effect, partly because they would have gone to therapy anyway:

*I don’t think it made any difference, because I was going to therapy anyway, so, I didn’t realise I was part of a study. Well it’s not that I didn’t realise it’s that I just, forgot about it. (female, 13)*

A further 12 young people stated that their participation in the study did not come up in therapy, and this seemed to imply that because it did not come up in therapy, it did not have an impact on their outcomes.

## Discussion

This study aimed to investigate adolescents’ experiences of participating in an RCT. More specifically, the aim was to explore three areas in relation to the participant experience: motivation to participate, whether or not participation was found to be acceptable and what affected this, and whether and how participation had impacted on their experience of therapy and therapeutic change.

Regarding reasons for participating, Ferguson [21] reported that most adult patients do not feel they have a ‘moral duty’ to agree to take part in clinical trials, whereas McCann, Campbell and Entwistle [22] suggest that decisions to do so are often altruistic. McCann et al. highlighted the willingness to contribute towards furthering scientific knowledge as a major reason, but noted that this is best understood as a form of ‘conditional altruism’, as patients also hope that they will benefit themselves. This is consistent with the findings of our study, where a significant proportion (43 %) suggested that helping others and contributing to wider understanding was their main motivation for participating in the trial. Other reasons (such as the hope of getting quicker access to treatment, or simply because the research sounded interesting) might indicate a hope for more personal gain, but as with the findings of McCann et al. [22] regarding adults, these different motivations are not contradictory, and it may well be that a form of ‘conditional altruism’ was also characteristic of most young people.

To what degree their consent to participate can be considered fully *informed*, however, is questionable, as considerable levels of confusion existed among our participants about what a randomised clinical trial entails. The proportion of those who demonstrated understanding (45 %) was hardly different from those who appeared not to understand (43 %), with specific problems reported by a fair number of adolescents regarding their understanding of the process of randomisation or the difference between what was being offered in the three treatment arms. These findings mirror the results of studies on adult samples with similar rates of the ‘therapeutic misconception’ [23]. In a qualitative interview study on middle-aged and older men with somatic prostatic diseases, most participants recalled major aspects of trial design, including the involvement of chance, but most also held other co-existing views about their treatment allocation like fate and individualised allocation [24]. A similar struggle to recall the details of the study was reported for a sample of patients on anti-psychotic medication [25]. In this study the confusion about trial processes and aims might have been due to difficulties recalling details of the informed consent that they were given weeks or months ago. Another possible explanation might be that some young people did not fully understand the study details in the first place, possibly as a result of their depression, which is often accompanied by difficulties with concentration [13].

Yet despite their limited understanding of the trial, having taken part in the IMPACT study the vast proportion of adolescents (93 %) found that it had been generally acceptable. In some cases this was because they had positively valued what taking part in the research had provided (e.g. helping them to reflect on progress or making them feel less isolated), but by far the greatest reason was because they had valued the relationships that they had formed with the research assistants who had undertaken the assessment meetings and were ‘the face’ of the study. The fact that these research assistants were friendly, flexible and engaging was key to the young people finding the study as a whole acceptable. Nevertheless, there were elements of the research with which they were not entirely happy. Whilst some elements were merely ‘awkward’ (such as the recording of therapy sessions), the greatest dissatisfaction was with the research questionnaires, which many young people felt to be overly time consuming, repetitive and in certain respects not meaningful. In some cases, this appears to have had a significant impact on the validity of the data produced, as some young people reported lacking the motivation to meaningfully respond to the questions.

These findings are consistent with studies examining the acceptability of trial design for adults in psychological therapy studies (e.g. [25–29]). For example, the young women with post-partum depression treatment in a study by Le et al. [28] spoke about finding questionnaires a helpful way to reflect on their own experiences. Furthermore, the young women formed relationships with the research team, which helped them to deal with feelings of isolation. Likewise, the young adults in a trial on social recovery CBT [4] generally reported quite high levels of acceptance regarding trial participation, although some aspects of the trial were criticized; for instance, some questions on the questionnaires were experienced as intrusive. Similar to the current study, the flexibility of the research assistants was an important aspect of acceptability.

The question of to what extent being part of the research impacted on the participants’ depression itself is a complex one, especially in a trial where the intervention is a ‘talking therapy’, which in some way has similarity to sitting down and speaking to a researcher. Our study suggests that a considerable minority of adolescents were confused about the difference between the research and the therapy or saw them as two aspects of the same thing. Interestingly, a considerable proportion of young people directly compared their experience of therapy with their meetings with the research assistants, sometimes preferring the latter because they were ‘friendlier’, ‘more flexible’ and the researchers were more like a peer than an adult. Clearly taking part in the research study did impact on their well-being—mostly in positive ways—such as reducing the sense of isolation or giving the young people an opportunity to reflect on the progress they had made. Although this is encouraging because it suggests that participating in an RCT of this sort was generally a positive experience for these young people, it does raise questions about the external validity of the findings and whether participating in the trial itself may have had a therapeutic impact.

### Implications of the study for clinical trials evaluating the effectiveness of psychological therapies with adolescents

Although caution is required in generalizing these findings to other research settings, our study suggests the following:

- As with adults, a range of reasons explain why the young people have agreed to take part, but in most cases, a form of ‘conditional altruism’ exists; that is, they feel that taking part will help other people, but they also hope that it may have a range of benefits for themselves such as accessing treatment faster or receiving a more high-quality intervention.
- Despite the under-representation of adolescents in clinical trials generally, when adolescents do take part in a clinical trial evaluating the effectiveness of psychological therapies, most young people generally find the experience acceptable.
- Factors that contribute to trial participation being acceptable include the opportunity to reflect on the changes they have made by routinely reviewing their progress and the fact that research assistants were friendly, flexible and worked hard to engage with the young people. Clearly, recruiting highly motivated research assistants and giving them appropriate training and support to undertake this task is essential if a trial of this sort is to be successful.
- Conversely, asking young people to fill in questionnaires that are repetitive or time consuming or that ask questions that do not seem relevant is likely to have a negative impact on their willingness to participate in clinical trials. Despite some concerns among psychotherapists, the recording of therapy sessions did not appear to be a major obstacle for the young people in the study.
- Despite the general acceptability, greater effort needs to be made to ensure that consent to participate is truly informed. In particular, helping young people to understand what the different interventions involve and how the process of randomisation takes place may lead to a more fully informed consent process in clinical trials. Considering the role of the member of the research team taking informed consent seems relevant here. Helping those young people who seem to be struggling with understanding, on a case by case basis, may be an important start. It may also be that information sheets are not always fit for purpose, as there can be a requirement to include large amounts of information, even at the expense of making the information engaging and accessible. Including young people in the design of information sheets and developing innovative methods of explaining studies, rather than relying on more traditional patient information sheets, may be an important element of achieving this. For example, using interactive smart technology such as tablets to deliver the study information and take informed consent might help young people to engage with the information more fully. Some concerns also exist about the external validity of trial findings in the light of the findings of this study. In particular, where questionnaires are felt not to be meaningful, young people may not be filling them in carefully or truthfully. Likewise, the fact that the young people reported quite positive experiences of meeting with research assistants to review their progress also suggests that elements of the research protocol may have themselves been therapeutic, potentially distorting the findings of the study regarding clinical effectiveness. More caution may be needed in interpreting the effects of RCTs of psychological therapies, which are often considered the gold standard form of assessing clinical effectiveness, given the potential impact of the method itself on treatment outcomes.

### Strengths and limitations of the study

The study reported here is one of the first to focus on the experience of young people taking part in a clinical trial evaluating the effectiveness of psychological therapies. The relatively large sample size, as well as findings based on in-depth interviews in which the young people had the opportunity to identify their own priorities and speak about what they considered to be most significant, add to the external validity of the study. This highlights the strengths and benefits of using qualitative research methods rather than pre-defined questionnaires or other methods of quantitative data collection.

Nevertheless, this study only included those who had already agreed to participate in the clinical trial, so some of the barriers to participation or reasons why young people might have chosen not to participate could not be identified. In addition, the adolescents who took part in the sub-study were only from one of the regions that were involved with the IMPACT study, so their experiences may not have reflected the experience of young people based in other regions, who also took part in this clinical trial. In addition, although the use of open-ended interviews had a number of benefits (especially in terms of allowing the young people to determine which aspects of participating in the study mattered to them), it did mean that the participants in the study were not all systematically asked about each element of trial participation (decision to join, understanding of the trial procedures, experience of participating, and impact on outcomes). For this reason, it was not always possible to give the exact proportions of young people who may have held various viewpoints. In particular, when topics were not mentioned by a particular participant, it is hard to say whether this reflects the fact that the topic was not important to them or whether they simply did not think to speak about it. As such, the current study is best seen as a preliminary exploratory study that has identified a range of experiences and views that adolescents could have when taking part in a clinical trial of this sort. Further research (e.g. using a standardized questionnaire) could build on this to systematically assess how common or uncommon such views are.

## Conclusions

This study highlights the importance of giving young people who participate in clinical trials the opportunity to report on their own experience of taking part. In doing so, there are opportunities to better understand what motivates young people to take part; what makes such participation more or less acceptable; and to explore the impact of the trial participation itself on the clinical outcomes being evaluated. This study suggests a number of factors that need to be considered when designing trials of psychological therapies with young people and suggests that caution must be taken in the interpretation of trial outcomes, given the impact of the research framework itself.

## Abbreviations

BPI, Brief Psychosocial Intervention; CAMHS, Child and Adolescent Mental Health Service; CBT, cognitive behavioural therapy; ETI, Expectations of Therapy Interview; FA, framework analysis; IMPACT, Improving Mood through Psychoanalytic and Cognitive-Behavioural Therapy; RCT, randomised controlled trial; STPP, short-term psychoanalytic psychotherapy
